# Editorial: Lipid metabolism and human diseases

**DOI:** 10.3389/fphys.2022.1072903

**Published:** 2022-11-04

**Authors:** Peter U. Amadi, Hong-Mei Gu, Kai Yin, Xian-Cheng Jiang, Da-wei Zhang

**Affiliations:** ^1^ Department of Pediatrics, Group on the Molecular and Cell Biology of Lipids, University of Alberta, Edmonton, AB, Canada; ^2^ Center for Diabetic Systems Medicine, Guilin Medical University, Guilin, China; ^3^ SUNY Downstate Health Sciences University, Brooklyn, NY, United States

**Keywords:** low-density lipoprotein (LDL), metabolic-associated fatty liver disease, coronary artery ectasia, idiopathic pulmonary fibrosis (IDF), coronary artery ectasia (CAE), atherosclerotic cardiovascular disease (ASCVD)

The metabolism of lipids is crucial to several functional processes in the body, including the storage of energy, regulation of hormones, and transportation of nutrients. These processes are disrupted when lipids become dysregulated, leading to lipid disorders. The Research Topic, Lipid Metabolism and Human Diseases, presents a critical insight into the latest advances and progress made in cardiovascular disease research. The Research Topic featured eight original research articles and four reviews that advanced the understanding of how lipid metabolism contributes to metabolic disorders.

The biology of lipoproteins and their roles in the progression of cardiovascular disorders is well documented. Low-density lipoprotein (LDL) plays a central role during cardiovascular homeostasis and primarily mediates the initiation and progression of atherosclerotic cardiovascular disorders (ASCVD). LDL cholesterol (LDL-C) is the main biological marker for LDL and remains the clinical target for ASCVD treatment, despite the fact that ASCVD risks persist in some patients with moderate LDL-C levels (Cromwell et al., 2007). LDL-C stands for cholesterol content in LDL particles (LDL-Ps). Qiao et al., in their article, exhaustively discussed the preference and precision of LDL-Ps over LDL-C in the prognosis of ASCVDs. The prognostic accuracy of LDL-C is affected by variations in lifestyle and drug intervention among individuals, which is why traditional lipid-lowering drugs like statins significantly reduce LDL-C levels and less of LDL-P levels. The article by Qiao et al. further outlined the atherogenic mechanisms of the action of LDL by focusing on subclasses of LDL-Ps, including sdLDL and ox-LDL, summarized the analytical techniques used for their measurement, and examined the advances in using statins and PCSK9i as LDL-lowering therapies.

In addition to elucidating the molecular mechanisms of proteins and pathways relevant to lipid metabolism, this Research Topic also covered the metabolic roles of lipids in the pathogenesis of metabolism-associated fatty liver disease (MAFLD) (Jia et al.), Alcohol-Associated Fatty Liver (Ferdouse and Clugston), Coronary Artery Ectasias (Liu et al.), Idiopathic Pulmonary Fibrosis (Geng et al.), and Prurigo Nodularis (Chu et al.). MAFLD, previously known as non-alcoholic fatty liver disease, occurs after significant accumulation of fats in the liver without any clear cause, like alcohol. Accumulation of triglycerides, impaired lipid metabolism, liver fibrosis and cirrhosis, non-alcoholic steatohepatitis (NASH), and in some cases, hepatocellular carcinoma are some common outcomes of MAFLD (Yki-Jarvinen et al., 2021). Of importance, MAFLD has been shown to share similar pathophysiological mechanisms with diabetes; however, establishing a common biomarker for both comorbidities has been arduous and inconclusive. Jia et al., in their study, have shown the suitability of monocyte to high-density lipoprotein cholesterol ratio (MHR) as a novel inflammatory biomarker. Through a rigorous and detailed study, they have established that T2DM patients with higher MHR have higher predisposition to be diagnosed with MAFLD.

Ferdouse and Clugston, in their article, proffered mechanistic insights on the mechanisms leading to ALD initiation, with a particular focus on hepatic lipid accumulation and the development of fatty liver. The article also strengthened the current understanding of how alcohol abuse impairs hepatic lipid metabolism. Lipid metabolism was reviewed in several transgenic models, including peroxisome proliferator-activated receptor γ (PPARγ) transgenic mice, and knockout mouse models of sterol regulatory element-binding protein 1 (Srebp1c), stearoyl–CoA desaturase-1 (SCD1), diglyceride acyltransferase (DGAT), and perilipin-2 (PLIN2). The article further highlighted the limitations associated with possible gender-based bias in earlier studies of AFD and other limitations like the use of low-fat/high carbohydrate Lieber-DeCarli (LDeC) liquid diet that may affect hepatic lipid accumulation pathways, and inducing whole body ablation of specific genes that may affect whole body lipid and hepatic lipid metabolism instead of liver specific knockouts.

Distinguishing the progression of coronary artery ectasia (CAE) from coronary artery disease (CAD) has been subject to extensive probing without any notable progress (Chou et al., 2022). However, Liu et al., in their study, used a targeted metabolomics approach to establish fatty acid biomarkers with high diagnostic performance for CAE progression from CAD. The study identified 35 promising metabolites of AA, EPA, and DHA that showed significant differences between CAE and CAD, out of which five of these biomarkers namely; 12-hydroxyeicosatetraenoic acid (12-HETE), 17(S)-hydroxydocosahexaenoic acid (17-HDoHE), EPA, AA, and 5-HETE, after rigorous screening showed the highest specificity for the diagnosis of CAE.

Idiopathic pulmonary fibrosis (IPF) is a fatal fibrotic disorder with no known cause (Sartiani et al., 2022). Geng et al. provided new insights into the role of fatty acid metabolism in the development of pulmonary fibrosis. The article provided extensive insights and expert opinions on the profibrotic processes that occur in distinct fibroblasts, macrophages, epithelial and lung cells. Aberrant fatty acid metabolism in the lungs and the contributory mechanisms leading to the overproduction of profibrotic lipids were well elucidated. The study further discussed the development of apoptosis and pro-fibrotic phenotypes in lung epithelial cells, and provided insights into the links between dysfunctional fatty acid metabolism and increased ER stress, macrophage polarization, and cytokines-induced fibroblast differentiation into myofibroblasts. In addition, some promising therapies that target the fatty acid metabolic pathway in idiopathic pulmonary fibrosis, and their possible mechanisms of action were well elucidated. Prurigo nodularis (PN), another chronic disease with unknown etiology, was also investigated by Chu et al. to understand how the severity of PN affects the levels of steroids. Using liquid chromatography-tandem mass spectrometry, they quantified the levels of cortisol, cortisone, testosterone, progesterone, and dehydroepiandrosterone (DHEA) in PN patients, matched to a control group. The study consistently showed a relationship between the levels of cortisol and cortisone and the severity of PN. This is the first study to demonstrate this relationship, therefore further studies can now target these biomarkers to achieve higher diagnostic accuracy for monitoring PN progression.

The study of Cao et al. investigated if the distribution of fatty acids in the sebum underpins the severity of acne among adolescents. It is well documented that inflammatory response and sebum production become elevated during acne. Earlier studies validated the hypothesis that variations in fatty acid distribution account for the inflammatory responses during acne (Zouboulis et al., 2014). However, until the study of Cao et al., the FA alterations in facial sebum remained to be clearly studied. The findings of the study showed the likelihood of the higher incidence of acne in females compared to males, may be due to the unique differences in fatty acid alteration in the facial sebum. Different anatomical sites in adult females showed altered fatty acid composition that inflamed the environment, mimicking the U-zone acne. These findings indicate that the development of gender- and site-specific therapeutic approaches for acne can target the levels of these specific fatty acids.

In conclusion, this Research Topic broadly covered various aspects of lipid metabolism and the consequences of impaired lipid metabolism. Expert insights were provided on the various pathophysiological mechanisms involved in the initiation and progression of different diseases associated with lipid metabolism, and in addition, current gaps in existing studies were highlighted. This Research Topic will thus become relevant in advancing both the current and future directions in providing new therapies for lipid disorders and the development of new diagnostic markers for lipid disorders.
